# DNA and sRAGE circulation in the early phase after polytrauma

**DOI:** 10.1186/cc14394

**Published:** 2015-03-16

**Authors:** P Joly, C Massé, D Dwivedi, P Liaw, J Marshall, Y Berthiaume, E Charbonney

**Affiliations:** 1 University of Montreal, QC, Canada; 2 McMaster University, Hamilton, ON, Canada; 3 University of Toronto, ON, Canada; 4 CRHSCM-University of Montreal, QC, Canada

## Introduction

Various DAMPS, alarmins are released after trauma. The soluble receptor for advanced glycation endproducts (sRAGE) was reported to be associated with acute renal failure and duration of ventilation [1]. Cell-free DNA (cfDNA) has been associated with prognosis in trauma patients [2]. We studied the kinetics of these two biomarkers over the first 5 days, in a cohort of severely ill trauma patients.

## Methods

Patients who had sustained serious traumatic injury, within 24 hours of trauma, were recruited in a level I trauma center. We collected ISS, baseline demographic characteristics, resuscitation information and daily organ dysfunction (MOD) scores, over 10 days. Blood samples were collected within 24 hours of trauma (day 0) and on days 1, 3 and 5. sRAGE was measured by ELISA and cfDNA was measured by UV absorbance after plasma isolation.

## Results

**Figure 1:**
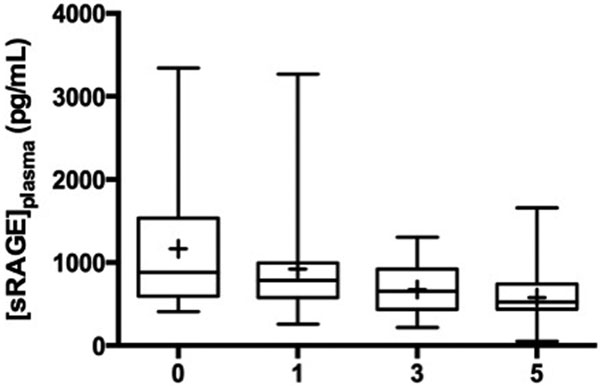
**sRAGE kinetics (days)**.

Median ISS was 39 and mortality was 21% (8/38). During the first 5 days after trauma, the median concentration of sRAGE (Figure 1) decreased significantly over time (*P *< 0.0001) while median levels of DNA did not (*P *= 0.73), and remained elevated compared with normal control. No correlation was found with ISS. Patients initially in shock had lower levels of sRAGE or cfDNA (*P *< 0.05) and had received more fluid (10.6 l vs. 5.25 l) or blood (6 l vs. 0.5 l). Day 3 and day 5 sRAGE levels were inversely correlated with PRBC received. Medians of sRAGE on days 0 (1,301 vs. 730 pg/ml) and day 1 (925 vs. 760 pg/ml) were significantly higher in nonsurvivors (*P *< 0.01). Finally, day 0 sRAGE was correlated with the maximal (*r *= 0.44; *P *= 0.007) and the cumulative renal failure component of the MODS, over the 10 days (*r *= 0.48; *P *= 0.005).

## Conclusion

DNA and sRAGE kinetics differ following trauma. Early elevation of sRAGE predicts mortality in univariate analysis and correlates with subsequent renal failure.
